# Role of a Putative Alkylhydroperoxidase Rv2159c in the Oxidative Stress Response and Virulence of *Mycobacterium tuberculosis*

**DOI:** 10.3390/pathogens11060684

**Published:** 2022-06-14

**Authors:** Gunapati Bhargavi, Amit Kumar Singh, Anbarasu Deenadayalan, Chinnaiyan Ponnuraja, Shripad A. Patil, Kannan Palaniyandi

**Affiliations:** 1 Department of Immunology, Indian Council of Medical Research-National Institute for Research in Tuberculosis, #1, Mayor Sathyamoorthy Road, Chetpet, Chennai 600031, India; bhargavi.gunapati@gmail.com (G.B.); harianbu@nirt.res.in (A.D.); cponnuraja@nirt.res.in (C.P.); 2 Indian Council of Medical Research-National JALMA Institute for Leprosy & Other Mycobacterial Diseases, Agra 282001, India; dramit.icmr@gmail.com (A.K.S.); shripadpatil@yahoo.com (S.A.P.)

**Keywords:** *Mycobacterium tuberculosis*, tuberculosis, alkylhydroperoxidases, Rv2159c, oxidative and transition metal stress response, virulence

## Abstract

*Mycobacterium tuberculosis*, which causes tuberculosis, is one of the leading infectious agents worldwide with a high rate of mortality. Following aerosol inhalation, *M. tuberculosis* primarily infects the alveolar macrophages, which results in a host immune response that gradually activates various antimicrobial mechanisms, including the production of reactive oxygen species (ROS), within the phagocytes to neutralize the bacteria. *OxyR* is the master regulator of oxidative stress response in several bacterial species. However, due to the absence of a functional *oxyR* locus in *M. tuberculosis*, the peroxidase stress is controlled by alkylhydroperoxidases. *M. tuberculosis* expresses alkylhydroperoxide reductase to counteract the toxic effects of ROS. In the current study, we report the functional characterization of an orthologue of alkylhydroperoxidase family member, Rv2159c, a conserved protein with putative peroxidase activity, during stress response and virulence of *M. tuberculosis*. We generated a gene knockout mutant of *M. tuberculosis* Rv2159c (MtbΔ2159) by specialized transduction. The MtbΔ2159 was sensitive to oxidative stress and exposure to toxic transition metals. In a human monocyte (THP-1) cell infection model, MtbΔ2159 showed reduced uptake and intracellular survival and increased expression of pro-inflammatory molecules, including IL-1β, IP-10, and MIP-1α, compared to the wild type M. tuberculosis and Rv2159c-complemented MtbΔ2159 strains. Similarly, in a guinea pig model of pulmonary infection, MtbΔ2159 displayed growth attenuation in the lungs, compared to the wild type *M. tuberculosis* and Rv2159c-complemented MtbΔ2159 strains. Our study suggests that Rv2159c has a significant role in maintaining the cellular homeostasis during stress and virulence of *M. tuberculosis*.

## 1. Introduction

*Mycobacterium tuberculosis* (*M. tuberculosis*), the causative agent of tuberculosis (TB) remains as one of the most successful human pathogens. The World Health Organization (WHO) reported about 10 million active cases and 1.5 million deaths due to TB in 2020 [1]. Following inhalation of infectious aerosol, *M. tuberculosis* is engulfed by alveolar macrophages into a phagosome, which is transported to endocytic compartments for further processing. In optimally activated macrophages, phagosomes with bacteria fuse with lysosomes and get killed by the cytolytic actions of the phagolysosomes [2]. However, *M. tuberculosis* utilizes various mechanisms to prevent phagosome–lysosome fusion, and to survive inside the macrophage [3]. Following phagocytosis, the host cells generate oxidative stress (OS), which is a consequence of imbalance in the regulation of reactive oxygen species (ROS) and the antioxidant mechanisms that detoxify the ROS [4]. During infection, the host system produces various ROS conditions. During infection, *M. tuberculosis* produces various peroxynitrites (ONOO^−^), hypochlorites (HClO), and peroxides (H_2_O_2_) enzymes to hydrolyze the host-derived chemicals that produce toxic ROS. *OxyR* and *soxR* are the major prokaryotic peroxide stress regulators of many pathogenic bacteria. However, *M. tuberculosis* lacks the *oxyR* system. Therefore, in *M. tuberculosis*, the peroxidase stress is mainly regulated by the orthologues of alkyl hydroperoxidase reductase (AhpC), which is a major peroxide sensor and transcriptional regulator in several pathogenic bacteria [5,6]. However, in other mycobacterial species, such as *M. leprae* and *M. avium*, *oxyR* controls the transcription of AhpC [7].

Bacterial AhpC is structurally similar to the eukaryotic antioxidant proteins with peroxidase activity, and reduces peroxynitrites and peroxides radicals generated by host cells during infection [8,9]. Overexpression of peroxidase enzymes enables *M. bovis* to resist killing by isoniazid, a first-line anti-TB drug [10]. The *M. tuberculosis* AhpC is a member of peroxiredoxin family proteins with conserved active cysteine residues at the substrate binding sites. For functional peroxidase activity, the AhpC should couple with a non-homolog substrate protein, AhpD and balance NADH or NADPH levels [11]. Similarly, AhpC is involved in the reactivation of dormant *M. tuberculosis*, which contributes to the transmission of infection into a new host [12]. Since AhpC protects *M. tuberculosis* against oxidative stress during progressive in vitro and in vivo infections, they are considered to be potent virulent determinants [13]. However, our understanding of the functional role of AhpC during intracellular survival of *M. tuberculosis* remains incomplete. Therefore, in the current study, we focused on the functional characterization of a conserved peroxidase encoded by Rv2159c, which is reported to be an orthologue of alkylhydroperoxidase family, and is predicted to be involved in defense mechanism of *M. tuberculosis*. Rv2159c has carboxymucanolactone decarboxylase domain that is mainly responsible for the peroxidase activity. In addition, presence of cysteine residues at the catalytic site (Cys-Pro-Try-Cys) of Rv2159c contributes to peroxidase activity. Enzymatic assays of the catalytic site indicate that Cys84 is a key residue for alkylhydroperoxidase activity of Rv2159c [14]. A protein-BLAST analysis revealed that Rv2159c shares amino acid sequence identity with similar proteins in other mycobacterial species, including *M. angelicum* (100% identity) and *M. riyadhense* (79.4% identity). However, expression of Rv2159c is significantly higher in *M. tuberculosis* compared to *M. bovis* virulent strain [15]. In addition to its peroxidase activity, Rv2159c interacts with PknI, which is one of the serine-threonine protein kinases responsible for cellular homeostasis in *M. tuberculosis* [16]. Earlier studies have reported that the interaction of Rv2159c with PknI occurs at the Ala49 and Gly50 amino acid residues [17]. Rv2159c has also been reported to interact with Rv0148, a putative short chain dehydrogenase involved in intermediary metabolism, homeostasis, virulence, and drug resistance of *M. tuberculosis* [18,19]. Gene knockdown studies on Rv2159c report reduced in vitro survival of the bacteria and a higher level of sensitivity to oxidative stress, compared to the wild type strain [14]. These findings suggest that Rv2159c has a potential role in the virulence of *M. tuberculosis*.

In the present study, we report the construction of knockout mutant of Rv2159c in *M. tuberculosis* H37Rv (MtbΔ2159) using specialized transduction procedure. The in vitro studies confirmed that the deletion of Rv2159c altered the growth profile of *M. tuberculosis*. Exposure of MtbΔ2159 to oxidative stress and transition metals displayed an impaired bacterial survival. The survival of MtbΔ2159 was also altered during macrophage infection. The secretion pattern of IL-1β, IP-10, and MIP-1α cytokines by infected macrophages suggest a pathogenic role for Rv2159c by affecting the host immune responses during *M. tuberculosis* infection. Further, guinea pig lung infection studies demonstrated that MtbΔ2159 was attenuated for growth, and resulted in reduced gross pathology in the lungs and spleen. Overall, the current study report that Rv2159c is a potent virulence factor involved in protecting the *M. tuberculosis* against oxidative damage and facilitating uptake and intracellular survival and pathogenesis during in vitro and in vivo infections, respectively.

## 2. Results

### 2.1. Construction of Mtb∆2159 Mutant in M. tuberculosis

The knockout mutant of *M. tuberculosis* lacking Rv2159c was constructed by a phage-based specialized transduction as mentioned in the methods section. In our approach, targeted gene deletion of 733 bp from the 5′ end of Rv2159c, rendered the functional motifs of Rv2159c defective and non-functional (Figure S1A,B). The 50-kb phAE159 low copy phasmid was chosen for phage propagation and further phAE159 and AES-digested products were ligated in phAE159 using Pac-I site and confirmed AES retained size of 6.587 kb (Figure S2A–D). After transduction, three clones were obtained and the colonies were screened by PCR using gene-specific primers of (i) hygromycin forward and right arm reverse (ii) left arm forward and hygromycin reverse primers; the product size was confirmed. Among the three constructs, Sanger sequencing was performed for one clone along with hygromycin sequence and the mutant was named as Mtb∆2159 (Table S1). The sanger sequencing data were deposited to data bank with a reference number 10.5281/zendo.6561038.

### 2.2. Growth Kinetics of Mtb∆2159

**Figure 1:**
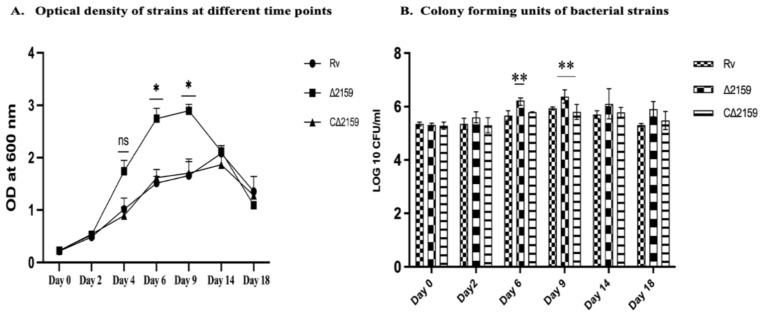
Growth kinetics of wild type H37Rv, MtbΔ2159, and CΔ2159 strains in liquid media. (**A**). Optical density of strains. The growing cultures were observed for optical density using spectrophotometer at specific time intervals at day 0 to day 18 and the graph was plotted. Two-way ANOVA with Bonferroni post-test was used and significance of * *p* < 0.1 was reported. (**B**) Colony forming units of bacterial strains. Number of bacterial CFUs were calculated per/mL of total culture and graphs were plotted. The experiment was performed in triplicates and the mean ± standard deviation values of the three independent experiments was used for the graph. Two-way ANOVA with Bonferroni post-test correction and the significance was reported by comparing with H37Rv. ** *p* < 0.01.

To determine whether the gene knockout of Rv2159c in *M. tuberculosis* brought any changes in the bacterial growth, we estimated CFUs as a measure of growth profiles and compared it between wild type H37Rv, Mtb∆2159, and CΔ2159 strains. As shown in Figure 1 we observed a significantly elevated growth of mutant (*p* < 0.01) compared to wild type H37Rv and CΔ2159 at 6 and 9 days of incubation. However, there was no significant difference observed between these three bacterial strains at day 14 and day 18 of in vitro growth (Figure 1A,B).

### 2.3. Viability of Mtb∆2159 during H_2_O_2_ Stress

**Figure 2:**
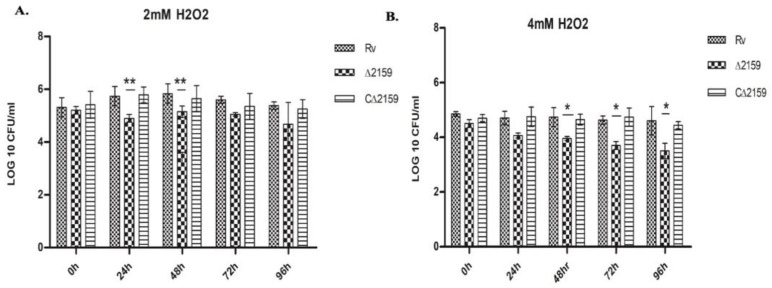
Effect of oxidative stress on the growth of MtbΔ2159. (**A**). CFU enumeration of *M. tuberculosis* strains treated with 2mM H_2_O_2_. The graphs representing log10 CFUs of wild type H37Rv, MtbΔ2159 and CΔ2159 strains treated with 2 mM H_2_O_2_ displaying sensitivity per/mL of total culture. (**B**). CFU of *M. tuberculosis* strains treated with 4 mM H_2_O_2_. The bar graphs represent the log10 CFUs of strains treated with 4 mM H_2_O_2_, the CFU data are presented per ml of total culture. Two-way ANOVA was using Bonferroni post-test was employed to identify the significance between the strains. Error bars indicate mean ± SD and significant at ** significant at *p* < 0.01 and * *p* < 0.1.

Since Rv2159c belongs to the alkylhydroperoxidase family, we were interested in validating the response of the mutant strain toward H_2_O_2_ stress (Figure 2A,B). The wild type *M. tuberculosis* H37Rv, Mtb∆2159, and CΔ2159 strains were treated with 2 mM and 4 mM H_2_O_2_ for up to 96 h. Our results show that exposure to 2 mM H_2_O_2_ significantly reduced the number of Mtb∆2159 CFU, compared to the wild type H37Rv, and CΔ2159 strains at 24 and 48 h post exposure. The growth of all the strains further declined after treatment with 4 mM H_2_O_2_. A significantly reduced number of Mtb∆2159 CFU was observed, compared to the wild type H37Rv, and CΔ2159 strains at 48, 72, and 96 h post exposure to 4 mM H_2_O_2_. These results suggest that Mtb∆2159 is more susceptible to oxidative stress, than wild type H37Rv, when treated with H_2_O_2_, in a concentration-dependent manner.

### 2.4. Effect of CuSO_4_ and ZnSO_4_ Treatment in Mtb∆2159

**Figure 3:**
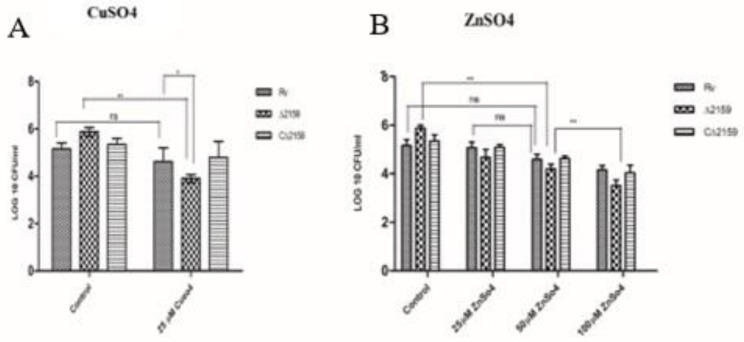
Effect of transition metal stress in MtbΔ2159. (**A**). Effect of 25 µM CuSO_4_. The mid-log phase cultures were plated onto 7H10 media supplemented with 25 µM CuSO_4_. The bar graphs represent the survival of bacteria in the presence and absence of CuSO_4_. (**B**). Effect of ZnSO_4_. The bar graphs represent the survival of wild type H37Rv, MtbΔ2159, and CΔ2159 strains after plating onto 7H10 media supplemented with 25 mM, 50 µM, and 100 µM ZnSO_4_. The statistical analysis was performed between treated strains of wild type H37Rv and MtbΔ2159. The bar graphs were plotted with the mean ± standard deviation values of three independent experiments carried out in triplicates. Two-way ANOVA with Bonferroni post-test correction was used for statistical significance calculation. The error bars indicate mean ± SD. ** *p* < 0.01 and * *p* < 0.05.

Transition metals such copper (Cu) and zinc (Zn) are part of several key enzymes, such as zinc-metallopeptidases and electron transport chain reactions in *M. tuberculosis* [20]. However, these metals can be toxic to *M. tuberculosis* at elevated levels present inside the infected host cells [21]. Therefore, the metal efflux detoxification system of *M. tuberculosis* needs to be regulated efficiently for bacterial survival inside the host cells [22]. To understand the role of Rv2159c during transition metal stress, by exposure to high levels of Cu and Zn, we treated the wild type H37Rv, Mtb∆2159, and CΔ2159 strains to various concentrations of transition metals and measured the bacterial survival. We noted a significantly reduced survival of Mtb∆2159, compared to wild type H37Rv and CΔ2159 upon exposure to 25 µM of CuSO_4._ We also analyzed the CFUs of wild type H37Rv, Mtb∆2159, and CΔ2159 strains after exposure to 25, 50, or 100 µM of ZnSO_4_ and observed that the mutant had a significantly reduced survival at 50 and 100 µM, compared to untreated controls (Figure 3A,B). Next, we exposed the wild type H37Rv, Mtb∆2159 and CΔ2159 strains to cell wall perturbing detergent, sodium dodecyl sulphate (SDS). We observed that although Mtb∆2159 was more sensitive to treatment with 0.01% SDS, compared to the wild type H37Rv and CΔ2159, difference was not statistically significant (Figure S3). The statistical analysis for these assays was calculated between the strains treated with chemicals and no-treatment controls.

### 2.5. Survival of Mtb∆2159 in THP-1 Cells

**Figure 4:**
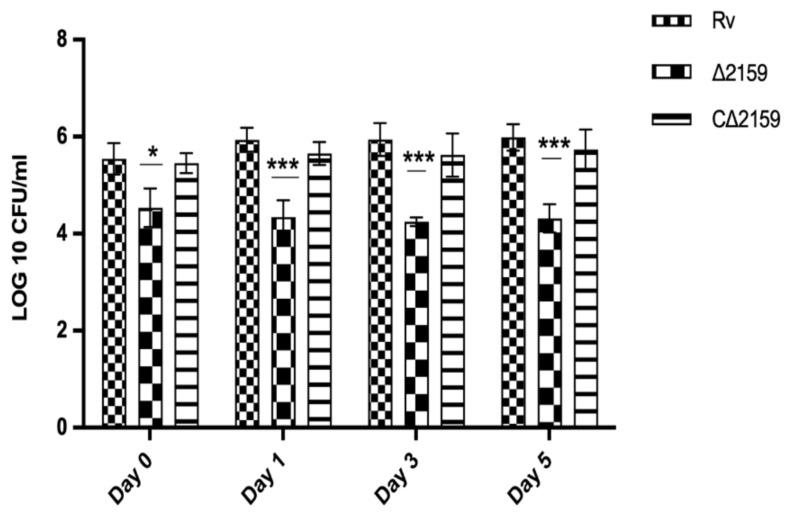
THP-1 macrophage infection of MtbΔ2159. The in vitro survival of wild type H37Rv, MtbΔ2159 and CΔ2159 strains was enumerated after post-infection on day 0, 1, 3, and 5. The bar graphs of wild type H37Rv, MtbΔ2159, and CΔ2159 log10 CFUs were plotted using the mean ± standard deviation values of three independent experiments carried out in triplicates. Two-way ANOVA with Bonferroni post-test correction was used for statistical significance calculation. *** *p* < 0.001 and * *p* < 0.05.

The uptake and intracellular survival of wild type H37Rv, Mtb∆2159, and CΔ2159 strains was assessed in a THP-1 macrophage infection model. As shown in Figure 4, the CFU of Mtb∆2159 was reduced at day 0 (i.e., 4 h post infection), with a statistical significance of *p* < 0.05. In addition, on day 1, 3, and 5 post infection, the intracellular growth of the mutant strain was significantly reduced compared to wild type H37Rv (*p* < 0.001) (Figure 4).

### 2.6. Cytokine Expression

**Figure 5:**
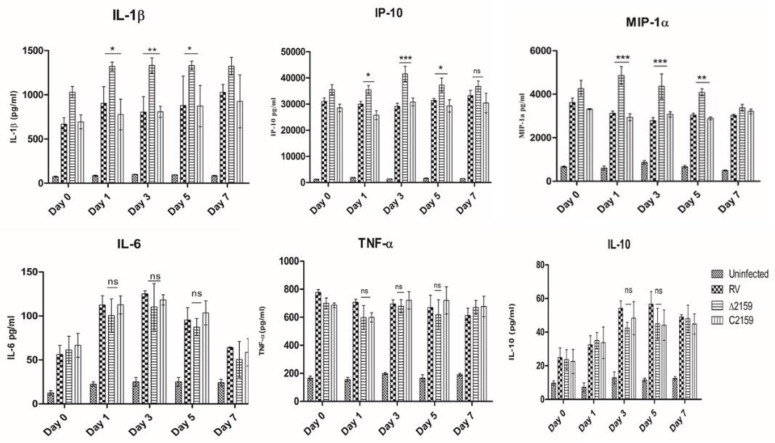
Cytokine levels during macrophage infection. Cytokine levels were measured at day 0, 1, 3, 5, and 7 post infection. Data were analyzed using two-way ANOVA. Experiment was performed using triplicates and graphs were plotted by considering mean values and error bars indicate mean ± SD. *** *p* < 0.001, ** *p* < 0.01, and * *p* < 0.05.

The cell-free supernatants of H37Rv, Mtb∆2159, and CΔ2159-infected THP-1 cells at days 0, 1, 3, 5, and 7 post infection were assessed for the expression of cytokines involved in various host immune functions. An increase in the levels of IL-1β, IP-10, and MIP-1α cytokines was detected in the Mtb∆2159-infected THP-1 macrophages, compared to the wild type H37Rv and C∆2159. We compared the individual cytokine expression levels at different time points and observed that compared to the wild type H37Rv, expression of IL-1β in Mtb∆2159 was increased by 1.5-folds on day 1, 3, and 5 post infection; IP-10 expression was increased by 1.5-folds on day 3; MIP-1 α expression increased by two-folds on day 1 and 3. The significance between wild type H37Rv and Mtb∆2159 for IL-1β, IP-10, and MIP-1α cytokines were statistically significant (*** *p* < 0.001, ** *p* < 0.01, and * *p* < 0.05) (Figure 5). No significant difference was observed in the expression of tested cytokines between wild type H37Rv and C∆2159-infected samples.

### 2.7. Mtb∆2159 Is Attenuated for In Vivo Growth in a Guinea Pig Model of Infection

**Figure 6:**
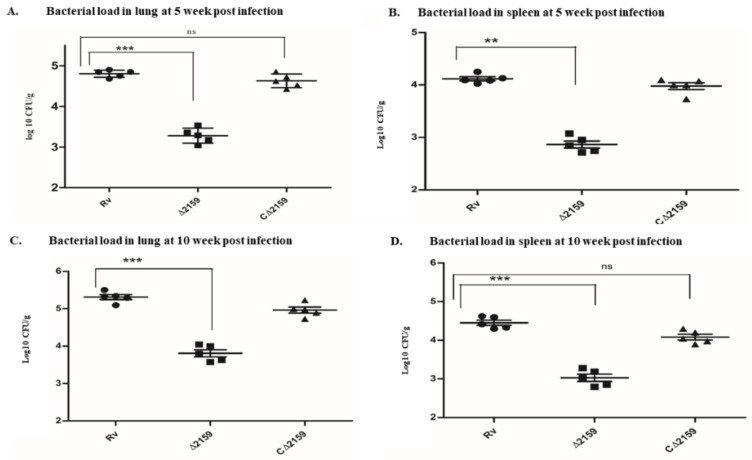
(**A**–**D**). Bacterial load of MtbΔ2159 at 5 and 10 week post infected lung and spleen. (**A**). Lung bacillary load of wild type H37Rv, MtbΔ2159, and CΔ2159 strains at 5 week post-infection. (**B**). Spleen bacillary load at 5 week post-infection of wild type H37Rv, MtbΔ2159, and CΔ2159 strains. (**C**). Lung bacillary load of wild type H37Rv, MtbΔ2159, and CΔ2159 strains at 10 week post-infection. (**D**). Spleen bacillary load of wild type H37Rv, MtbΔ2159, and CΔ2159 strains at 10 week post-infection. The graphs were plotted with the mean ± standard deviation of five independent animals in each group. Statistical analysis was performed using Two-way ANOVA with Bonferroni post-test correction. *** *p* < 0.001 and ** *p* < 0.01.

After observing the change in in vitro growth kinetics and altered intracellular survival of Mtb∆2159 in THP-1 cells, we wanted to determine the virulence of this mutant during in vivo infection. For this, we choose a guinea pig aerosol infection model (Figure 6). At five week post-infection, we observed reduced number of lung lesions in the mutant-infected group, compared to the wild type H37Rv or C∆2159-infected animals (Figure S4). At five week post-infection, the bacterial load in the lungs of Mtb∆2159, wild type H37Rv and C∆2159 were 2.7 log10 CFU, 4.4 log10 CFU, and 4.3 log10 CFUs, respectively. Thus, the bacillary load in the mutant-infected lungs was significantly reduced by 1.6 logs, compared to wild type H37Rv-infected lungs (Figure 6A). Similarly, at five week post-infection, the bacterial load in the spleen of Mtb∆2159, wild type H37Rv, and C∆2159-infected guinea pigs corresponds to 2.5 log10 CFU, 4.2 log10 CFU, and 4.1 log10 CFU, respectively, with about 1.8 log10 CFU reduction in the spleen bacterial burden in the Mtb∆2159-infected animals, compared to the wild type H37Rv-infected animals (Figure 6B). There was no significant difference in lung or spleen bacterial load between the wild type H37Rv and C∆2159-infected guinea pigs.

At ten week post-infection, the inflammation and gross pathology in lungs and the spleen was less severe in the Mtb∆2159-infected guinea pigs, compared to the wild type H37Rv and C∆2159-infected guinea pigs (Figure S5). The in vivo survival of Mtb∆2159, wild type H37Rv and C∆2159 in the lungs at ten week displayed 3.5 log10 CFU, 5.3 log10 CFU, and 5 log10 CFU, respectively, with a 1.8 folds reduction in bacterial load in the mutant compared to wild type H37Rv-infected animals (Figure 6C). Further, bacterial load of spleen at ten week post-infection in the Mtb∆2159, wild type H37Rv and C∆2159 infected animals corresponds to 2.9 log10 CFU, 4.5 log10 CFU, and 4.3 log10 CFU, respectively, with the mutant (Mtb∆2159) displaying a 1.6 folds reduction in in vivo survival, compared to the wild type H37Rv-infected animals (Figure 6D). The bacterial load was low in the spleen compared to the lung in all the tested groups at 5- and 10-weeks post-infection. The complemented strain (C∆2159) displayed bacterial burden similar to the wild type H37Rv-infected guinea pigs, with high pathological damage and lesions, compared to Mtb∆2159-infected group (Figure 6A–D). Thus, deletion of Rv2159c has diminished the intracellular survival and virulence of *M. tuberculosis* in the lungs and spleen of infected guinea pigs.

## 3. Discussion

*M. tuberculosis* resists host-derived oxidative stress through a network of closely associated antioxidant enzymes. Superoxide detoxifying enzyme (SodA), an integral membrane protein (DoxX), and thiol-oxidoreductase (SseA), catalase/peroxidase (KatG), alkylhydroperoxide reductase (AhpC), peroxiredoxin (AhpE), and thioredoxin reductase (Tpx) are crucial for the in vitro and in vivo survival of *M. tuberculosis* [23]. SOD acts as a superoxide radical scavenger that converts the virulent-associated superoxides to molecular oxygen (O_2_) and H_2_O_2_. It also senses the accumulation of hydrogen peroxide [24,25]. Likewise, DoxX and SseA are involved in thiol oxidoreductase activity and confers resistance to the bacteria against oxidative stress mediated by thiol radicals. Moreover, it is reported that DoxX interacts with SseA in the absence of SodA [26].

Apart from these detoxification enzymes, AhpC, AhpD, and AhpE are playing an important role in the bacterial defense against ROS [27]. AhpE is a cysteine peroxiredoxin; considered as a potential drug target against *M. tuberculosis* [28]. These enzymes are crucial in the adaptation of *M. tuberculosis* to the hostile stress conditions generated by the host during infection. Therefore, the alkyl hydroperoxidase system is considered as key for the virulence of *M. tuberculosis*. Previous studies in *M. bovis* suggest that knockdown of *ahpC* gene attenuated the bacterial virulence in a guinea pig infection model [12]. Hence, studies on alkylhydroperoxidases and their related genes would help to understand their functional role in *M. tuberculosis* pathogenesis.

In order to examine the role of Rv2159c during bacterial cell division, we compared the bacterial growth profiles and observed that the mutant displayed surge in growth, compared to the wild type and complemented *M. tuberculosis* strains. The increased growth of Mtb∆2159 suggested a significant role for this gene during the growth of *M. tuberculosis*. Accordingly, we expected that a change in growth might affect the colony morphology of the mutant. However, we did not observe any difference in the colony morphology of the mutant, compared to the wild type and complemented strains.

Mycobacterial species mainly rely on the transcriptional regulator *oxyR* to survive during stress conditions generated by ROS. However, *oxyR* is disrupted to a non-functional state in pathogenic *M. tuberculosis*, which leads to the reliance of the bacteria on various other antioxidant bacterial genes. Indeed, expression of several of such antioxidant genes, including AhpC, is induced upon exposure of *M. tuberculosis* to ROS-generating agents [6]. Thus, the loss of a functional *oxyR* did not affect the pathogenic potential of *M. tuberculosis* during in vitro as well as in vivo conditions [28]. In this study, we report the potential contribution of an alkylhydroperoxidase gene (Rv2159c) to mycobacterial resistance against ROS and for successful *M. tuberculosis* uptake and intracellular survival.

Through DNA sequence analysis, we found that the Carboxymucanolactone decarboxylase domain (CMD) region involved in the ROS and oxidative stress was deleted in our knockout mutant strain (Mtb∆2159). The exposure of mutant to oxidative stress displayed a higher sensitivity to H_2_O_2_. The sensitivity of Mtb∆2159 to 2 mM and 4 mM H_2_O_2_ correlated with studies on *ahpC* mutant of *M. smegmatis*, which displayed enhanced sensitivity to exposure to oxidative stress generating agents [29,30]. Our results are also supported by earlier studies on the mutants of *ahpC* in *E. coli*, which were hypersensitive to H_2_O_2_ exposure, and the survival of bacteria was inhibited by organic hydroperoxides treatment [30]. In the current study, we mainly focused to validate the response of mutant toward oxidative stress, and hence the experiment was conducted using H_2_O_2._ Our findings strongly suggest that Rv2159c is involved in maintaining the redox homeostasis of *M. tuberculosis*.

Peroxidases mediate oxidation reaction when exposed to transition metal ions such as copper, zinc, manganese, cobalt, and lead [31,32]. Bacterial species such as *M. tuberculosis* protect themselves from metal toxicity by regulating the uptake of metals from the host cells during intracellular survival [33]. During infection, the host system generates metal-poisoning strategies that inhibit *M. tuberculosis* replication. Since the bioavailability of copper and zinc are scarce, the bacteria relies on various membrane transporters to uptake these metals for intracellular survival during infection [34]. In this study, we tested the toxicity of copper and zinc, which are essential micro nutrients involved in intracellular killing of *M. tuberculosis* [21,22]. We observed that exposure to copper and zinc had a significant impact on the survival of Mtb∆2159, suggesting a role for Rv2159c in the survival of *M. tuberculosis* against these metal-induced stress conditions. Further mechanistic studies are needed to fully understand the functional role of Rv2159c in mediating resistance to Cu, Zn, and other metals.

The uptake and intracellular survival of Mtb∆2159 was impaired in THP-1 macrophages, compared to the parental and complemented *M. tuberculosis* strains. These results are substantiated by earlier studies on the *ahpC* deletion mutant of *M. tuberculosis* during macrophage infection, which showed a similar reduction in the intracellular survival of the mutant [34]. We further analyzed the cytokine expression profile of THP-1 macrophages during Mtb∆2159 infection. The enhanced expression of proinflammatory cytokines IL-1β in Mtb∆2159-infected THP-1 cells suggested that Rv2159c has a role in regulating inflammation during *M. tuberculosis* infection. It is also probable that the attenuated growth of Mtb∆2159 during THP-1 infection is due to the higher expression of IL-1β cytokine [35,36]. Consistently, a previous study has reported that the secretion of proinflammatory cytokines has a key role in the defense against *M. tuberculosis* [37]. We also measured the level of other pro-inflammatory cytokines, such as IL-6 and TNF-α, and observed no significant difference between Mtb∆2159 and wild type H37Rv-infected host cells. In contrast, a higher level of expression of interferon-γ inducible protein (IP-10) [38,39], MIP-1α (Macrophage Inflammatory Protein) [40] chemokines were noted in THP-1 cells infected with Mtb∆2159, compared to the wild type *M. tuberculosis*. These observations suggest that Rv2159c has a selective and important role in regulating the host immune responses during infection, which appears to be independent of IL-6, TNF-α but associated with the induction of IL-1β, IP-10, and MIP-1α. Future studies should address the mechanistic association between Rv2159c expression and the regulation of these host immune molecules.

Several studies have reported that alkylhydroperoxidase family members play a significant role in in vivo survival and pathogenesis of bacteria such as *Helicobacter*, *M. tuberculosis,* and *M. bovis* [8,41,42,43]. Based on the findings on *ahpC* mutants that differentially affected the cytokine expression and showed reduced survival under stress conditions, we predicted that Rv2159c might regulate the virulence of *M. tuberculosis* in vivo. We used a guinea pig model of pulmonary aerosol infection to examine the virulence of Mtb∆2159, in comparison to the wild type and complementing *M. tuberculosis* strains. Guinea pigs are considered a better animal model than mice to study TB pathogenesis, since the former model closely mimics the disease pathology, including the formation of necrotic lung granulomas, as seen in human TB patients [44]. This model has also been used previously to evaluate oxidative stress response and redox sensing mechanism of *M. tuberculosis* [45,46]. In the current study, we observed considerably reduced bacillary load in the lungs and spleen of guinea pigs infected with Mtb∆2159 compared to the wild type H37Rv and complemented *M. tuberculosis* strains. The differential bacterial load also correlated well with the gross pathology of lesions noted in guinea pig organs infected with Mtb∆2159, wild type H37Rv, and complemented *M. tuberculosis* strains. Together, these observations confirm that Rv2159c is involved in in vivo survival and virulence of *M. tuberculosis*.

## 4. Conclusions

Overall, the current work shows that *M. tuberculosis* Rv2159c is a potent virulence factor that enable the bacteria to survive during stress conditions. Deletion of Rv2159c impaired the growth of *M. tuberculosis* in macrophages, and induced the expression of pro-inflammatory cytokines, suggesting that Rv2159c is involved in regulating host-immune responses. The role of Rv2159c in the in vivo pathogenesis of *M. tuberculosis* was confirmed through guinea pig model of infection. Based on our findings, we propose that Rv2159c is involved in maintaining redox homeostasis and virulence of *M. tuberculosis*.

## 5. Materials and Methods

### 5.1. Plasmids, Bacterial Strains, and Growth Conditions

The *Escherichia coli* (*E. coli*) DH5α (Invitrogen, Waltham, MA, USA) and HB101 (Takara, Kusatsu, Japan) cells used for cloning experiments were grown in Luria Bertani (LB) broth (Hi-Media, Mumbai, India). Middlebrook 7H9 broth (Difco, Franklin Lakes, NJ, USA) supplemented with 0.05% Tween-80 and 0.2% glycerol was used to grow *Mycobacterium smegmatis* (mc^2^155). *Mycobacterium tuberculosis* H37Rv was also grown in 7H9-OADC-Tween media supplemented with hygromycin 50 µg/mL, wherever required and kanamycin 25 µg/mL. Details of oligonucleotide primers, constructs, and plasmids used in the current study are defined in (Table S2).

### 5.2. Construction of Four Fragment Ligation

Rv2159cis a peroxidase gene of *M. tuberculosis*, and is 1032 bp in size. The protein product encoded by Rv2159c possess a carboxymucanolactone decarboxylase domain between amino acids 46 and 126, corresponding to 138–378bp. No other functional domains are present in the rest of the amino acid sequence of this protein. The deletion of Rv2159c was carried out using upstream ~1000 bases flanking left arm of Rv2160c and downstream right arm Rv2158c sequence regions. The amplified regions were ligated and cloned onto an antibiotic-resistant cassette of p0004-SacB plasmid for homologous recombination. The recombinant clones were screened using the Van91I restriction site; this enzyme identifies a discontinuous palindrome sequence interjected by five bases of sequence. The restriction enzyme digestion analysis confirmed clones were labelled as four fragment ligation or allelic exchange substrate (AES) and named as pGB2159 [18,47].

### 5.3. Construction of Transducing Phages Using AES

The phAE159 temperature-sensitive shuttle phasmid carrying Pac-I restriction site was further used for cloning. Allelic exchange substrate was cloned into phAE159 using Pac-I and transduced into *E. coli* HB101 competent cells along with in vitro packaging extract, to increase the efficacy of recombinant clones. The resultant transduced phasmid (pGB2159a) was purified and incorporated into *M. smegmatis* to recover high-titer phages at 30 °C with MP buffer (1 M Tris pH 8, 5 M NaCl, 1 M MgCl_2_ and 1 M CaCl_2_).

### 5.4. Specialized Transduction

*Mycobacterium tuberculosis* was grown to mid log phase (OD_600_ 0.5 to 0.8). The culture was centrifuged at 4000× *g*, and the pellet was resuspended in MP buffer and infected with high-titer phages. The cells were incubated at 37 °C overnight for phage augmentation and centrifuged at 4000× *g* for 10 min at 37 °C. Transduced *M. tuberculosis* cells were grown on 7H10 Middlebrook agar (Becton, Dickinson, Franklin Lakes, NJ, USA) containing 150 µg/mL of hygromycin. Five colonies were randomly chosen for screening and confirmed by gene-specific PCR and Sanger sequencing using hygromycin cassette as the target for the forward primer and RHS reverse primer; left arm forward and hygromycin reverse primer as to confirm transduction. The mutant was named as Mtb∆2159.

### 5.5. Complementation of Mtb∆2159

The coding region of Rv2159c gene (1032 bp) was amplified and ligated onto mycobacterial integrative vector pMV361 after being digested with restriction enzymes *EcoRI* and *Hind-III*. The resultant clones after PCR confirmation were further electroporated into Mtb∆2159 strain and transformants were selected using kanamycin 20 µg/mL on 7H10 agar plates. The PCR confirmed complement of Mtb∆2159 was labelled as C∆2159, and used for performing in vitro and in vivo assays.

### 5.6. Growth Kinetics Analysis

The log-phase cultures of wild type *M. tuberculosis* H37Rv, Mtb∆2159, and C∆2159 strains were grown in Middlebrook 7H9-OADC-Tween media (Becton, Dickinson) with 180 rpm shaking at 37 °C. At various time points (days 0, 2, 6, 9, 14, and 18), 100 µL aliquot of cultures were taken to determine bacterial growth kinetics. Bacterial numbers in the cultures of wild type H37Rv, Mtb∆2159, and C∆2159 strains were assessed by spotting serially diluted cultures in 7H9 broth onto 7H10 OADC agar media without Tween-80. Number of bacterial colony forming units (CFU) was calculated after 3–4 weeks of incubation period of the plates at 37 °C. The total bacterial number was expressed as CFU per ml of the original culture. The Mtb∆2159 and C∆2159 strains that were confirmed by sequencing was used for all the characterization studies reported in the current work.

### 5.7. Colony Morphology Analysis

The Mtb∆2159 morphology was assessed by comparing the colonies with wild type H37Rv and C∆2159 strains. Bacterial strains were grown in 7H9 broth and 20 µL of each culture was spotted onto 7H10 OADC agar plates without Tween-80 and with/without hygromycin as needed. The colony morphology was analyzed manually after incubation of plates at 37 °C for 3–4 weeks.

### 5.8. Growth Analysis during Exposure to H_2_O_2_, Transition Metals, and SDS

The wild type *M. tuberculosis* H37Rv, Mtb∆2159, and CΔ2159 strains were grown in 7H9-OADC-Tween-80 media supplemented with appropriate antibiotics and exposed to different concentrations of hydrogen peroxide (H_2_O_2_) as described previously [28], and the number of bacterial CFUs was determined at 0, 24, 48, 72, and 96 h post-exposure. The CFUs were performed by plating the serially diluted bacterial cultures onto 7H10 OADC agar media and plates were incubated for 3–4 weeks at 37 °C.

### 5.9. Exposure to Transition Metals and SDS Stress

To determine the bacterial response to exposure to transition metals and SDS, wild type *M. tuberculosis* H37Rv, Mtb∆2159, and CΔ2159 strains were spread onto 7H10 agar plates containing 25 µM CuSO_4_; 25, 50, and 100 µM of ZnSO_4_, or 0.01% SDS as reported by Vandal et al., 2009 [48]. The untreated bacterial strains were used as control. Growth of untreated and treated bacteria was monitored and calculated as CFU after 3–4 weeks of incubation at 37 °C.

### 5.10. THP-1 Cell Line Infection

The in vitro survival of wild type *M. tuberculosis* H37Rv, Mtb∆2159, and C∆2159 strains was analyzed using THP-1 monocyte-derived macrophage infection model. THP-1 cell lines were obtained from NCCS (Table S2) and cell lines were grown in Roswell Park Memorial Institute (RPMI) medium (Gibco, Waltham, MA, USA) supplemented with 10% fetal bovine serum (Thermo Scientific, Waltham, MA, USA). The cell counts were determined using trypan blue assay. Briefly, 10 µL of the cells was mixed with 5 µL of trypan blue stain and 185 µL RPMI and mixed gently by pipetting. From this cell suspension, 10 µL aliquot was placed into a hemacytometer and viable cells that excluded trypan blue were counted. For infection experiments, 1 × 10^6^ cells were seeded onto 24-well tissue culture plates, and differentiated into macrophages using 50 mM phorbol 12-myristate 13-acetate (PMA); the cells were incubated for 48 h at 37 °C in the presence of 5% CO_2_. The macrophages were washed with fresh RPMI, further incubated overnight, and infection was performed with wild type *M. tuberculosis* H37Rv, Mtb∆2159, and C∆2159 in triplicate wells with a 1:10 (bacteria: macrophages) multiplicity of infection (MOI). Infection was allowed for 4 h (*t* = 0), and extracellular bacteria were eliminated by treating the infected cells with streptomycin at 1 µg/mL for 4 h [49]. The infected cells were washed off the media and lysed with sterile water followed by pipetting. The viability of intracellular bacteria was estimated by plating the serially diluted lysates of infected THP-1 cells onto 7H10 OADC agar on day 0, 1, 3, and 5 post infection. The plates were incubated for 3–4 weeks at 37 °C for colony formation. The CFU was calculated and presented as per mL (i.e., per well) of the lysate.

### 5.11. Cytokine ELISA

The THP-1 cells were infected with wild type *M. tuberculosis* H37Rv, Mtb∆2159, and CΔ2159 strains at an MOI of 0.1. The infected cell-free supernatants were recovered on days 0 (4 h), 1, 3, 5, and 7 post infection to determine the level of cytokines IL-1β, IL-6, IL-4, IP-10, IL-10, MIP-1α, and TNFα using Cytokine ELISA Kit according to the manufacturer’s protocol (BD, Pharmingen, San Diego, CA, USA). Briefly, the captured antibody (100 µL) was added to the plate and incubated overnight. Antibody was aspirated from the wells and plates were blocked with 200 µL of assay diluent, incubated for 30 min at room temperature (RT), and washed with phosphate-buffered saline containing Tween20 (PBS-T). Standards and samples (100 µL) were added to the antibody-coated plate and incubated for overnight at 4 °C followed by washing with PBST. Then, the detection antibody and enzyme reagent (100 µL) were added to the wells and incubated for 1 h at RT, followed by washing with PBST. Further, 100 µL of substrate solution was added to the wells and incubated till color developed, and the reaction was stopped by adding 50 µL of stop solution. The plates were analyzed using Spectra Max 250 ELISA reader (Molecular Devices, San Jose, CA, USA) with absorbance range of 450 nm and λ correction wavelength of 570 nm. The curve fit was applied to each standard curve according to instructions provided by the manufacturer and sample concentrations were determined using a standard curve. The obtained values were validated, and significance was reported.

### 5.12. Infection Studies in Guinea Pigs

The Dunkin–Hartley strain of guinea pigs weighing 300–350 gm was maintained in the National JALMA Institute of Leprosy and Other Mycobacterial Diseases, Agra, India in a biosafety level III facility. Ten Guinea pigs in each group were infected with 50–80 bacilli of wild type *M. tuberculosis* H37Rv, Mtb∆2159, or CΔ2159 strains through aerosol route (Inhalation Exposure System, Glasscol Inc., Terre Haute, IN, USA). Ten animals without infection were used as control group. At 5 and 10 week post-infection, five animals from each infected and control groups were euthanized using thiopentone sodium (100 mg/kg body weight) (Neon Laboratories Ltd., Mumbai, India) injection via intraperitoneal route. After dissecting each guinea pig, the lung, spleen, and liver were observed for gross pathological changes. A portion of the lung lobe and spleen from the infected and control group was removed aseptically and homogenized in 5 mL saline by using a Teflon glass homogenizer. The homogenates were serially diluted using 7H9 broth and plated onto 7H10 plates in triplicates and incubated at 37 °C for 3–4 weeks. Bacterial colonies were enumerated and represented as log10 CFUs.

### 5.13. Statistical Analysis

The graphs represented in the manuscript were generated with data from three independent experiments carried out using triplicate samples. We used GraphPad Prism 5.0 (GraphStat Technologies, Bengaluru, India) to analyze and calculate the mean, standard error of the mean (SEM), or standard deviation of the data obtained from in vitro growth kinetics of *M. tuberculosis* strains, macrophage infection, H_2_O_2_, CuSO_4_, ZnSO_4_, SDS stress assays, cytokine analysis, and in vivo survival in the guinea pig lungs and spleen. To determine statistical difference between multiple groups, two-way ANOVA was performed with Bonferroni post-test correction method and significance was reported (* *p* < 0.05, ** *p* < 0.01 and *** *p* < 0.001).

## Data Availability

The data that support the findings of this study are available on request from the corresponding author.

## Supplementary Materials

The following supporting information can be downloaded at: https://www.mdpi.com/article/10.3390/pathogens11060684/s1, Figure S1: (A). Flow chart explaining the gene knockout construction of Rv2159c. (B). Confirmation of knockout mutant using right arm gene specific primers; Figure S2: Construction of gene knockout mutant; Figure S3: Effect of SDS stress in MtbΔ2159; Figure S4: Gross pathology of 5 week post infected lung and spleen of wild type H37Rv, MtbΔ2159 and CΔ2159 strains displaying lesions; Figure S5: A-B. Lung and spleen displaying lesions at 10 week post infection of wild type H37Rv, MtbΔ2159 and CΔ2159 strains; Table S1: Sanger sequencing of gene knockout mutant sequence displaying Hyg cassette and right arm amplified regions; Table. S2: Primers used in the current study and constructs of the study.
