# Supplementary material for: Role of a Putative Alkylhydroperoxidase Rv2159c in the Oxidative Stress Response and Virulence of Mycobacterium tuberculosis
Source: Pathogens. 2022 Jun 14;11(6):684. doi: 10.3390/pathogens11060684 (PMC9227533; doi:10.3390/pathogens11060684)
Supplement: Supplementary file 1 [file pathogens-11-00684-s001.zip › pathogens-1731554-supplementary.pdf]

Table S1. Sanger sequencing of gene knockout mutant sequence displaying Hyg cassette and right arm amplified regions

GCGCAGTTCCTCTGGGGGCCGCCGGACACCGCCCCCGGCGCCTGACGCCCCGGTCCTGTATCCTAAATCAAATATC
GGACAAGCAGTGTCTGTTATAACAAAAAATCGATTTAATAGACACACCAACAGCATGGTTTTTATGTGTGCGATAA
TTTATAATATTTCGGACAGGACTCTAGCCAAAGATTGGCCCCGCATCAGGTGACCGACGACGACGTCGCCGCGGC
CCGATCCCTGCTCGACACCGATGCGGCGCTGGTTGGCGCCCTGGCCTGGGCCGCCTTCACCGCCGCGCGGCGCAT
CGGCACCTGGATCGGCGCCGCCGCCGAGGGCCAGGTGTCGCGGCAAAACCCGACTGGGTGAGTGTGCGCGCCCT
GTCGGTAGGGTGTCATCGCTGGCCCGAGGGATCTCGCGGCGGCGAACGGAGGTGGCGACACACGTGGAGGCTG
CGCCCACTGGCTTGCGCCCAACGCCGTCGTGGCGTTCGGTGGCGGCACTGGCGATCAGTCGGCGCGGCCTGGCCG
AAGGTCCAGCTCAGCGTGCCGTCAACCCGCTGGCAGGTCGGCGCCGCCGACGCGGATGCCGATGGTGCCGATCA
GCCCGGCGACGCGTCCGGCAGCCCGTAACCCGGCCTCGACCAGATAGGTGGTGGTGGTCTTGCCGGACGTTCCG
GTGATCCCGATAACCGTCAACCGCTCGGACGGATGCCCGTACACGGTGGCGGCCAAGCCGCCGAGCACGCCGCG
GGGTGCGGGGTGCACCAACACGGGCACGGCCGCTCGTCCGGCGATCTCGGCGACCCCGGCGGGGTCGGTGAGC
ACCGCGACGGCGCCGCGTGCGATCGCGTCGCCGACGTGGCGGGCCCCGTGGGTGGTCGAGCCGGTCAGGGCGG
CGAACAGGTCACCGGGTGACACGTCCTGGGCGCGCAGCGTGACCCCGGTGACCGTCCGGTCCTCGGTGACGGCA
CGCTGAGCTGGACCCTCGGCCAGGGCCGCGCCGACCTGATCGGCCAGTGCGGCCAACCGAACGCCCACGACGGC
GTTGGGRCGCAAGCCRGTGGGCGCAGCCTCSACCTGTGKCGSCRCCTCCGTKCGCCGRCCGMGAGATCCCTCGGG
MCAGCRATGASRCSMCTACCGACAGRGGCGMGCRCCRCTCAGCMCWTGTCGKSGYYTWYGCCRCGACWGCCKR
GGTCCGKCTTGSGRKYGGACTSSGCMKGRTCMGGGTWCAGRSKSSGCCCCCTGGSSCSGRAGGGGKAASAGSYCT
CASGCCGTGGMMCYGTTSWCT

Intact Right arm region start site

Overview of upstream and downstream sequences of Rv2159c with primer marked regions

cgtcggtgccgccgtcgtgggcgtggccgaaaccggccgtggcgccgccggtgctgttcgggtgccggcccagtgcgcgggcgtt
cgaggtgatcgcccgccacggcgacggttggcagccgatcgaggggtacggcgagctcctgggcgcgttgccgatgctgcacgcc
gcgttcgagcgtgccgggcgagatccggcgaccgcccaggtctgtgtgtactcgtcggccggcgacccggcgaccctgcacgagt
accgccgggccggtgtcgcggaggtggcgctcgcgctgccctcggcgggccgcgaccaggtgctggccgccctggaccggtcggc
cccgctggtggatgcgttcgccggagacgaccgggaggtcaaaagccatgcctagcgccgacgtcggccgccaaacccgggcgca
gatcctgcgcgccgcgatggatatcgcctcggtgaaggggctctcggggctgtccatcggcgagcttgccgggcggctgggcatg
agcaagtcgggcctgttccggcatttcggcgccaaggagcagctgcagctggcgaccgtcgaggccgccgtgagcgtgttcgaag
ccgaggtcgtggctcccgcgatggcagcgccgcccggggtggaccgggtgcgcgccctcatgcatgcgtgggtcggatacctgga
acgcgacgtgccggcggctgctttttcgcggccgcggccgccgacgtggactcacagcctggcccggtgcgcgaccgcatcgccg
cgaccgggcgggccggaatcgccgccatcacggccgacgtcgaaacggcgcaacgccggggcgagatccgggcggatatcgaagt
gcgccaactcgcgttcgagctgcacgcctacgcgatggaggccaactgggcgctgctgctgctcgacgacgacggcgccggagag
cgggcgcgaacggcgatcgacgcggccctggccagagtcggcaccacccaggagggagtcgaatcatgaaatttgtcaaccatat
tgagcccgtcgcgccccgccgagccggcggcgcggtcgccgaggtctatgccgaggcccgccgcgagttcggccggctgcccgag
ccgctcgccatgctgtccccggacgagggactgctcaccgccggctgggcgacgttgcgcgagacactgctggtgggccaggtgc
cgcgtggccgcaaggaagccgtcgccgccgccgtcgcggccagcctgcgctgcccctggtgcgtcgacgcacacaccaccatgct
gtacgcggcaggccaaaccgacaccgccgcggcgatcttggccggcacagcacctgccgccggtgacccgaacgcgccgtatgtg
gcgtgggcggcaggaaccgggacaccggcgggaccgccggcaccgttcggcccggatgtcgccgccgaatacctgggcaccgcgg
tgcaattccacttcatcgcacgcctggtcctggtgctgctggacgaaaccttcctgccgggggcccgcgcgcccaacagctcat
gcgccgcgccggtggactggtgttcgcccgcaaggtgcgcgcggagcatcggccgggccgctccacccgccggctcgagccgcga
acgctgcccgacgatctggcatgggcaacaccgtccgagcccatagcaaccgcgttcgccgcgctcagccaccacctggacaccg
cgccgcacctgccgccaccgactcgtcaggtggtcaggcgggtcgtggggtcgtggcacggcgagccaatgccgatgagcagtcg
ctggacgaacgagcacaccgccgagctgcccgccgacctgcacgcgcccacccgtcttgccctgctgaccggcctggccccgcat
caggtgaccgacgacgacgtcgccgcggcccgatccctgctcgacaccgatgcggcgctggttggcgccctggcctgggccgcct
tcaccgccgcgcggcgcatcggcacctggatcggcgccgccgccgagggccaggtgtcgcggcaaaacccgactgggtgagtgtg
cgcgccctgtcggtagggtgtcatcgctggcccgagggatctcgcggcggcgaacggaggtggcgacacaggtggaggctgcgcc
cactggcttgcgccccaacgccgtcgtgggcgttcggttggccgcactggccgatcaggtcggcgcggccctggccgagggtcca
gctcagcgtgccgtcaccgaggaccggacggtcaccggggtcacgctgcgcgcccaggacgtgtcacccggtgacctgttcgccg
ccctgaccggctcgaccacccacggggcccgccacgtcggcgacgcgatcgcacgcggcgccgtcgcggtgctcaccgaccccgc
cggggtcgccgagatcgccggacgagcggccgtgcccgtgttggtgcaccccgcaccccgcggcgtgctcggcggcttggccgcc
accgtgtacgggcatccgtccgagcggttgacggttatcgggatcaccggaacgtccggcaagaccaccaccacctatctggtcg
aggccgggttacgggctgccggacgcgtcgccgggctgatcggcaccatcggcatccgcgtcggcggcgccgaccttcccagcgc
gctgaccaccccggaggcccccacgctgcaggcgatgctggcggcgatggtcgaacgcggggtggacaccgtggtcatggaggtg
tccagccacgcgctggcgctgggccgggtggacggcacccggttcgccgtcggcgccttcaccaatctctcccgtgaccacctgg
atttccaccccagcatggccgactacttcgaggccaaggcgtcattgttcgatccggactcggcactgcgcgcccgcaccgccgt
ggtgtgcatcgacgacgacgccgggcgcgcgatggcggcgcgggccgccgacgcgatcaccgtcagcgccgccgaccggcccgca
cactggcgcgccacggatgtggcgcccacggacgcgggcgggcaacaattcaccgccatc

Rv2160c

Deleted Region of Rv2159c

Rv2158c

Yellow highlighted is left arm primers

Blue highlighted is right arm primers

Grey highlighted is deleted region of Rv2159c gene replaced with 739 bp by hyg cassette

Continued

Table. S2: Primers used in the current study and constructs of the study.

Name of Primer	Sequence	Purpose
2159 RAF	TTTTTTTTCCATAGATTGGCCCCGCATCAGGTGACCGAG	Cloning
2159 RAR	TTTTTTTTCCATCTTTTGGGGGGCCTCCGGGGTGGTCAG	Cloning
2159 LAF	TTTTTTTCCATAAATTGGTTCGGCGCCAAGGAGCAGCTG	Cloning
2159 LAR	TTTTTTTTCCATTTCTTGGCCGGGGACAGCATGGCGAGC	Cloning
CΔ2159 Forward	CCAAGAAATGGAAAAAAAACGGGGACAGCATGGCGAGCG	Cloning
CΔ2159 Reverse	CCAAAAGATGGAAAAAAAAGGGGCCTCCGGGGTGGTCAG	Cloning

Plasmids & constructed strains in the current study

**Plasmids**	**Description**	**Reference/origin**
p0004- SacB	Suicide recombination delivery vector carrying *hyg*^{R}-*sacB* for gene disruption, *hyg*^{R}	Jain et al.,2014
phAE159	Conditionally replicating shuttle phasmid vector	Jain et al.,2014
pMV361	*E. coli* mycobacterial shuttle vector, *kan*^{R}, *hsp*60 promoter	Stover et al.,1991
pMV261	*E. coli* mycobacterial shuttle vector, *kan*^{R}, *hsp*60 promoter carrying 6x- His-tag (GTG-GTG-GTG-GTG-GTG-GTG)	Stover et al.,1991
**Constructs**		
pGB2159	p0004- SacB carrying the left and right arm fragments of Rv2159c gene from *M. tuberculosis*, *hyg*^{R} (four fragment ligation)	This study
pGB2159 a	phAE159 carrying four fragment ligation, *hyg*^{R}	This study
	Knockout strain constructed	This study

MtbΔ2159	in this study	
CΔ2159	Complement of Rv2159c gene from *M. tuberculosis* constructed in this study	This study
**Cell lines**		
THP-1 cell line	Human leukemia monocytic cell lines	National Centre for Cell Sciences (NCCS), Pune, India

Continued

Supplementary information

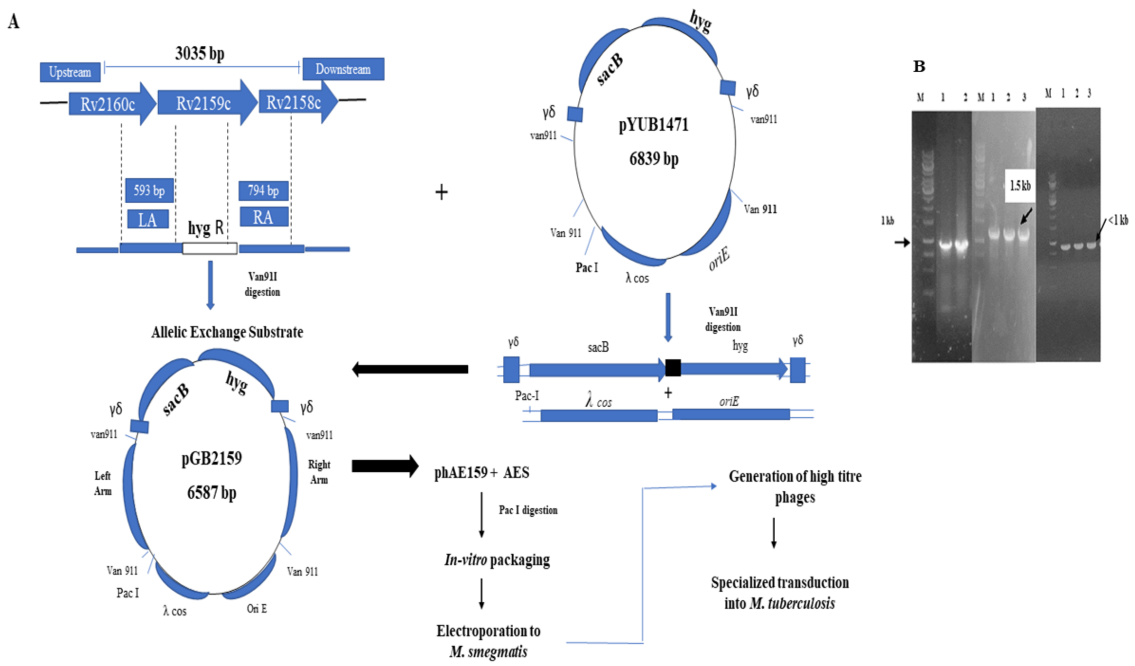

Figure S1. A. Flow chart explaining the gene knockout construction of Rv2159c. B. Confirmation of knockout mutant using right arm gene specific primers M; 1 kb ladder, Lane 1and 2 PCR amplification of Rv2159c right arm with 794 bp, M; 1 kb ladder, Lane 1to 3 amplification of knockout mutant with hygromycin forward and reverse primers displaying product size of 1.5 kb, M; 1 kb ladder, Lane 1and 2 PCR amplification of knockout mutant with left arm forward and hygromycin reverse primers displaying product size <1 kb.

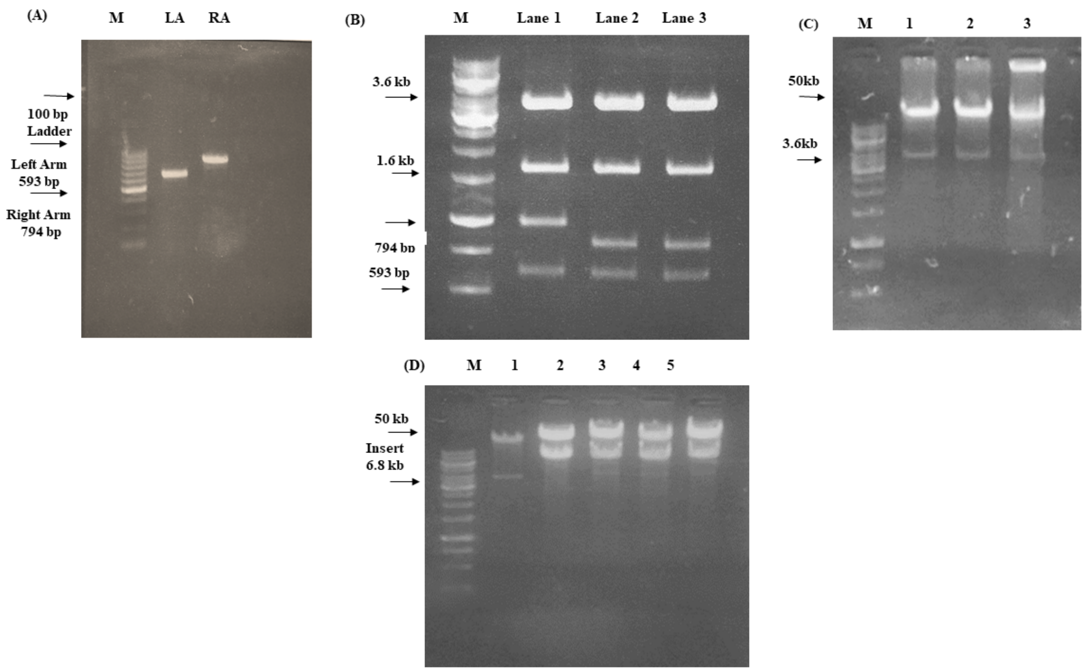

Figure S2. Construction of gene knockout mutant. A.PCR amplification of Rv2159c. M; 100 bp ladder, Lane 1; amplified left arm PCR of Rv2159c with 593 bp, Lane 2; right arm PCR amplified with 794 bp. **B. Confirmation of four fragment ligation** M; 1kb marker, Lane 1; SacB vector digested with Van 91I displayed 4 bands with 3.6 kb, 1.6 kb, 979 bp, 567 bp, Lane 2 & 3; recombinant clone of Rv2159c displaying 4 bands; 3.6 kb, 1.6 kb of SacB vector, left arm 593 and right arm 794 bp. **C. Confirmation of phAE159 digestion using Pac-I** M; 1 kb ladder, Lane 1 to 3 phAE159 digested with Pac-I displayed release of insert 3.6 kb. D. **Screening of recombinant containing four fragments ligation** M; 1 kb marker, Lane 1; PhAE159 digested with Pac-I taken as control, Lane 2 to 5; Recombinant clones digested with Pac-I release of 6.8 kb AES insert.

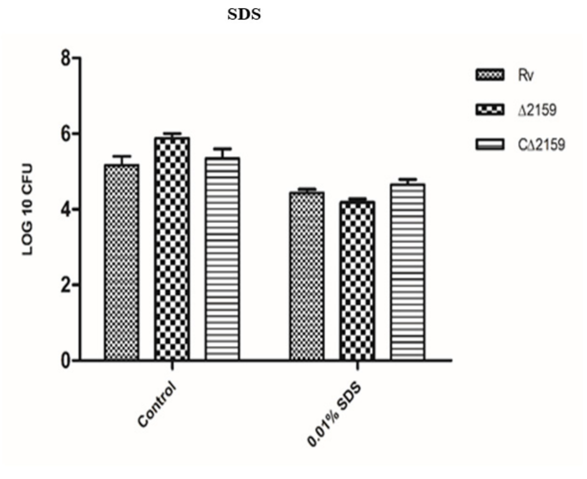

Figure S3. Effect of SDS stress in MtbΔ2159. The mid-log phase cultures were plated onto 7H10 media treated with 0.01% SDS. The bar graphs represent log10 CFUs of the survival of bacteria in the presence and absence of 0.01% SDS. Data represented in the graphs is the mean of three independent experiments carried out in triplicates and the bar graphs were plotted by taking the mean and standard deviation and analyzed using Two-way ANOVA with Bonferroni post-test correction and error bars indicate mean ± SD.

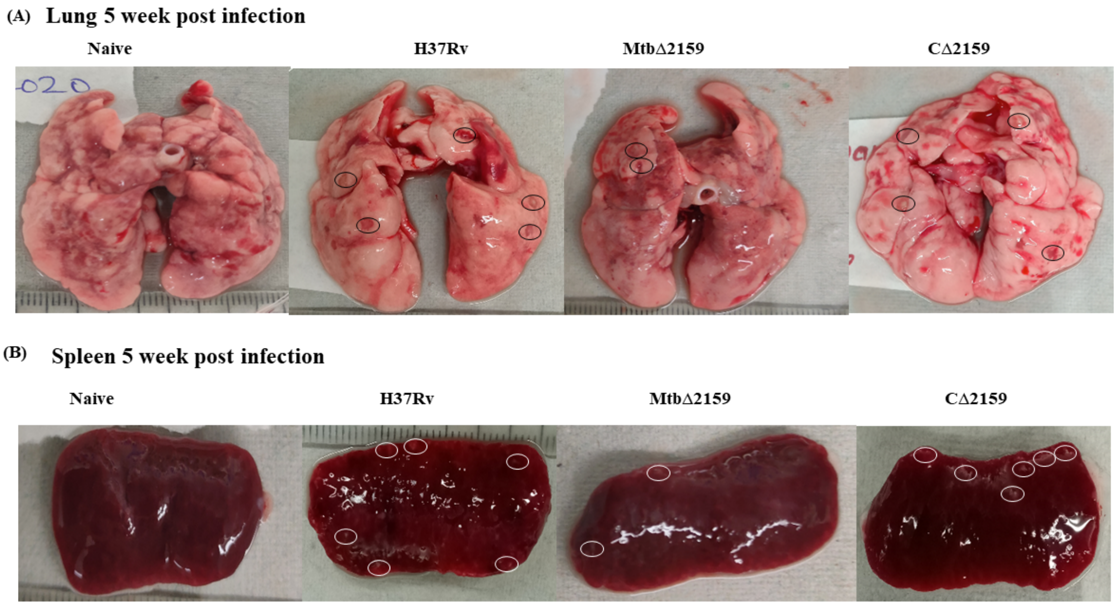

Figure S4. Gross pathology of 5 week post infected lung and spleen of wild type H37Rv, MtbΔ2159 and CΔ2159 strains displaying lesions. A. Guinea pigs infected lung with MtbΔ2159 resulted in fewer lung lesions compared to animals infected with wild type H37Rv. **B.** Gross pathology of 5 week post- infection spleen of H_{37}Rv, MtbΔ2159 and CΔ2159 strains**.** Guinea pigs at 10th week post-infection exhibited minimal tubercles compared to wild type H37Rv.

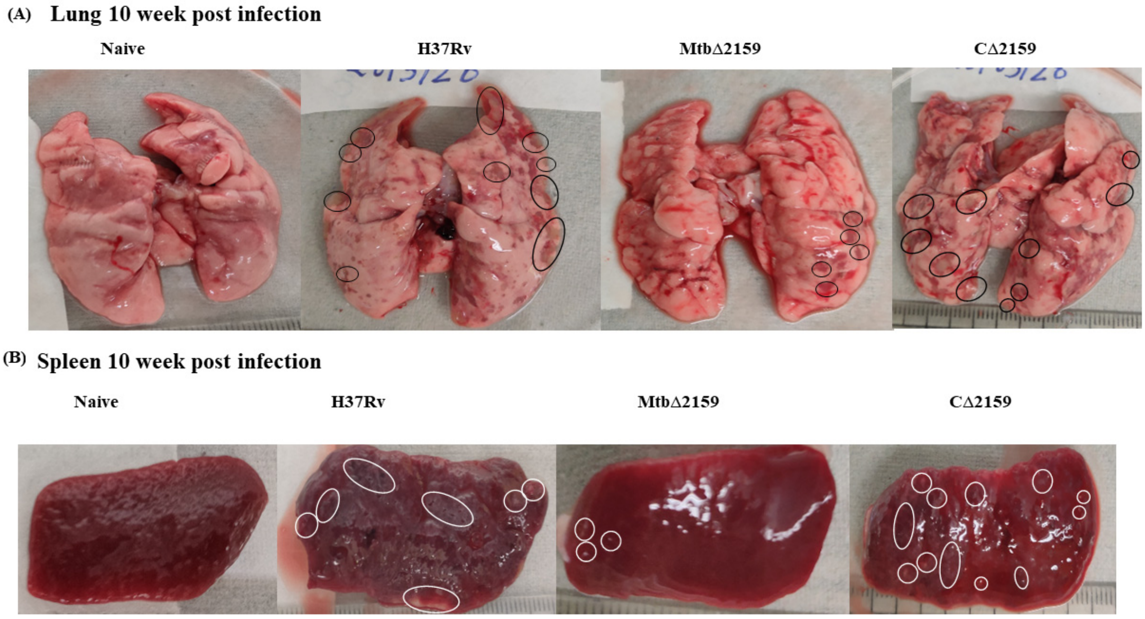

Figure S5. A-B. Lung and spleen displaying lesions at 10 week post infection of wild type H37Rv, MtbΔ2159 and CΔ2159 strains. **A.** The 10 week post infected lungs; n=5 of wild type H37Rv, MtbΔ2159 and CΔ2159 strains**.** The infected guinea pigs lung exhibited fewer lesions compared to that of wild type H37Rv with numerous lesions. **B.** Gross pathology of 10 week post infected spleen of wild type H37Rv, MtbΔ2159 and CΔ2159 strains. At 10 week, infected guinea pigs lung and spleen exhibited higher bacterial burden in wild type H37Rv compared to the mutant group.
